# Editorial: Model organisms in neuroinflammation and neuropathy: *Drosophila melanogaster*

**DOI:** 10.3389/fnagi.2024.1502502

**Published:** 2024-10-14

**Authors:** Zihan Huang, Xueji Dai, Baichun Jiang, Yan Kong

**Affiliations:** ^1^Department of Biochemistry and Molecular Biology, School of Medicine, Southeast University, Nanjing, China; ^2^Department of Genetics, School of Basic Medical Sciences, Cheeloo College of Medicine, Shandong University, Jinan, China

**Keywords:** neuroinflammation, neuropathy, *Drosophila* model, neurological disorder, aging

Neuroinflammation is the activated inflammatory response in the central nervous systems, including dysregulated cytokine release, reactive cytokine release, and activation of immune cells (Han et al., 2021). A plethora of evidence showed that neuroinflammation contributes to the pathogenesis of neurological disorders including Alzheimer's disease, Parkinson's disease, anxiety, and major depressive disorder (Leng and Edison, 2021; Sampson et al., 2016; Guo et al., 2023; Wu and Zhang, 2023). With evolutionally conserved mechanisms of immune response and convenience for genetic manipulation, *Drosophila melanogaster* is a widely used model organism to study the molecular mechanisms of neuropathy (Suzuki et al., 2023; Bussmann and Storkebaum, 2017; Muqit and Feany, 2002). To provide the overview of this Research Topic, we have selected four original research and review articles focusing on the neuroinflammation and neuropathy using *Drosophila melanogaster*.

In one research article, Zhao et al. investigated the role of total ginsenosides (TGGR) from ginseng on senescence. Feeding with TGGR during early adulthood extended the lifespan of *Drosophila melanogaster* dramatically. The food intake and reproductive capacity were not influenced by TGGR. Additionally, it improved the motility, intestine barrier, biorhythm homeostasis, and resilience to various stresses. Transcriptome profiling and molecular study showed that TGGR exert beneficial effects on longevity by targeting on genes involved in insulin, TOR and MAPK signaling. This study highlighted the translational value of active components in the traditional Chinese medicine on aging.

Dravecz et al. studied the contribution of Insulin/IGF-like signaling (IIS) to lifespan and health span. They previously found that targeted inhibition of neuronal IIS extended the lifespan. However, the negative geotaxis was unaffected and the exploratory walking behavior was worsened. This disconnection made them clarify the role of IIS in different types of neurons. They overexpressed the dominant negative form of insulin receptor to impair IIS in neuron type specific manner. Lifespan and locomotion abilities were used as redouts. Interestingly, IIS reduction selectively in serotonergic neurons extended the lifespan without influencing locomotion. In contrast, IIS inhibiton in cholinergic, GABAergic, dopaminergic, glutamatergic, and octopaminergic neurons had no effects or detrimental effects on lifespan and locomotor senescence. This study gave new ideas for neuron type-specific roles in lifespan and health span.

In a review article contributed by Asthana and Shravage, the role of mitophagy in Parkinson's disease *Drosophila* model was systemically discussed. As the second prevalent neurodegenerative disorder, Parkinson's disease is characterized as the degeneration of dopaminergic neurons, aggregation of α-synuclein (α-syn), and locomotor defects. Mitophagy is one of the major types of autophagy and regulates mitochondrial homeostasis. Impaired mitophagy was tightly correlated with PD. This review focused on the role of mitophagy modulators and potential PD drugs targeting mitophagy in *Drosophila* model. This review will accelerate the research of PD related mitophagy and targeting strategy in the future.

The mini-review by Bhattacharya shed light on peripheral neuropathy and axon degeneration. Traumatic, toxic, or genetically-induced insults impair the peripheral axons. They discussed the model establishment of peripheral nerve injury and neuropathy in *Drosophila*. The effects of injury on neurons and glias were systemically summarized. The peripheral axon injury models also gave platforms to clarify genes that participate in the pathogenesis and potential intervention strategies.

We expect the selected reviews and research articles could further our understanding of neuroinflammation and neuropathy. They also highlight the great value of *Drosophila melanogaster* as model organisms for the research of molecular pathogenesis and treatments in the field.
